# A Strategy for Suppressing Bundling in Dielectrophoretically Assembled Carbon Nanotube Arrays

**DOI:** 10.3390/nano16090512

**Published:** 2026-04-24

**Authors:** Kai Wang, Rongbin Xie, Jianze Xiao, Yingnan Yang, Chaoqun Li, Zhengming Hao, Xiao Lei, Wenshan Li

**Affiliations:** 1 State Key Laboratory of Micro-Nano Engineering Science, School of Mechanical Engineering, Shanghai Jiao Tong University, Shanghai 200240, China; 2 Micro-nano Engineering Sciences Research Center, School of Mechanical Engineering, Shanghai Jiao Tong University, Shanghai 200240, China

**Keywords:** dielectrophoresis, effective deposition region, carbon nanotubes, array assembly

## Abstract

Densely packed semiconducting carbon nanotube (CNT) arrays with well-controlled morphology are highly desirable for high-performance CNT-based electronics. Although dielectrophoresis (DEP) enables precise, efficient, and site-selective assembly, increasing array density often destabilizes process regulation and aggravates nanotube bundling because of the dynamic interplay among assembly conditions. Here, we introduce the effective deposition region (EDR) to reformulate DEP assembly into a framework that links DEP conditions and final arrays through an interpretable CNT deposition dynamic based on the effective DEP capture. Within this framework, experiments and modeling indicate a self-regulating, negative-feedback mechanism in which conductive CNT bridging reduces the gap voltage, contracts the EDR, and weakens sustained CNT-capture capability, thereby driving the assembly toward self-termination. By synergistically optimizing the applied voltage, electrode configuration, and CNT dispersion concentration to regulate EDR contraction, we obtained dense, bundle-suppressed CNT arrays with the number of nanotubes per unit width of approximately 140 tubes µm^−1^. The formation of small bundles implies that further combination of EDR-regulated assembly with additional inter-tube interactions is required to realize dense, monolayer CNT arrays. This work provides a coherent mechanistic framework for understanding feedback-regulated DEP assembly and enables a practical approach for optimizing both densification and morphology control in CNT array assembly.

## 1. Introduction

Semiconducting single-walled carbon nanotubes (CNTs) have emerged as one of the promising channel materials for post-silicon electronics due to their quasi-one-dimensional structure, high carrier mobility, near-ballistic transport, and compatibility with low-temperature integration processing [1,2,3,4,5,6]. To fully exploit these intrinsic advantages in large-area, high-performance integrated circuits (ICs), it is essential to organize CNTs into horizontally aligned, dense, bundle-free monolayer arrays. Such architectures can maximize on-state conductivity by providing parallel charge transport pathways while simultaneously minimizing off-state leakage [7,8,9].

Various strategies have been developed to fabricate CNT arrays, including direct growth by chemical vapor deposition (CVD) and post-growth solution assembly [10,11,12]. Although CVD can produce well-aligned arrays, it typically requires high growth temperatures and still faces substantial challenges in achieving wafer-scale uniformity and high semiconducting purity [13,14,15]. In contrast, solution-based routes are compatible with low-temperature fabrication and allow the utilization of pre-sorted, high-purity semiconducting CNT dispersions obtained through techniques such as polymer wrapping or density gradient ultracentrifugation [16,17,18]. Among these, dimension-limited self-assembly (DLSA) has enabled wafer-scale monolayer CNT arrays with tunable densities ranging from 20 to 300 tubes μm^−1^ [8,19]. However, DLSA relies on slow evaporation kinetics, which inherently limits its throughput and lacks precise site-selective assembly capability.

Dielectrophoresis (DEP), which exploits the interaction between induced dipole moments in CNTs and spatial gradients in a non-uniform electric field, is particularly attractive for its efficiency, flexibility, and site selectivity in CNT assembly [20,21,22]. A primary optimization target in DEP-based CNT array assembly is to increase array density while preserving favorable array morphology. In most previous studies, density improvement of DEP-assembled CNT arrays has been pursued mainly through a direct DEP-condition to array-optimization route, where DEP parameters such as the electrical signal, CNT concentration, and assembly time are tuned with respect to the final array morphology [23,24,25,26]. For instance, Sarker et al. tuned the array density from 1 to 25 tubes μm^−1^ by varying frequency and CNT concentration [27]. Shekhar et al. reported DEP-assembled arrays with densities of ~30 tubes μm^−1^ by adjusting the CNT concentration [28]. More recently, Liu et al. reported single-layer aligned CNT arrays assembled by DEP with a linear density of 54 ± 2 tubes μm^−1^ under prolonged assembly durations [29]. However, despite these advances in CNT array density, insufficient attention has been paid to the coupled effects of multiple DEP conditions and the dynamic evolution of the assembly process. Consequently, the further densification of array unavoidably comes at the expense of deteriorated morphology, particularly forming large bundles, which degrades device performance [8,30,31].

To address this issue, we introduce the effective deposition region (EDR) as a fundamental, intermediate and interpretable concept for understanding and regulating CNT assembly via DEPs, with the aim of mitigating bundling during array densification. In the DEP process, the formation of conductive CNT bridges between electrodes contracts the EDR and weakens sustained CNT capture, thereby offering feedback for regulating further assembly. Through the synergistic optimization of applied voltage, electrode configuration, as well as CNT dispersion concentration, we obtained bundle-suppressed and dense CNT arrays with approximately 140 tubes per unit width. A further reduction in residual bundles will likely require a combination strategy of EDR regulation and additional consideration of inter-tube interactions. These results illustrate that the EDR concept not only provides a useful framework for understanding the DEP assembly evolution, but also offers an interpretable route for fabricating high-quality horizontal CNT arrays.

## 2. Materials and Methods

### 2.1. Preparation of CNT Dispersions

Arc-discharged single-walled CNTs were purchased from Carbon Solution Inc., California, USA. The conjugated polymer [9-(1-octylonoyl)-9H-carbazole-2,7-diyl] (PCz) was obtained from Derthon Optoelectronics Materials Science Technology Co., Ltd, Shenzhen, China. A stock dispersion was prepared by mixing raw SWCNT powder and PCz in xylene at a mass ratio of 1:1 (*w*/*w*), resulting in a total solids concentration (CNTs + PCz) of 1 mg/mL. Then the mixture was subjected to sequential tip-sonication (Lab750, Sinap Tec, Lezennes, France) at amplitudes of 20%, 50%, and 40% for 10, 15, and 15 min, respectively, to promote polymer adsorption and CNT debundling. During sonication, the dispersion was intermittently cooled in an ice-water bath to suppress temperature rise. Subsequently, the dispersion was centrifuged at 60,000× *g* for 1 h (Beckman Optima XE-90 Ultracentrifuge, California, USA), and the upper 90% of the supernatant, enriched in high-purity semiconducting CNTs, was carefully collected for subsequent experiments. The typical optical absorbance spectrum of the CNT dispersions is provided in Figure S1.

### 2.2. Fabrication of Array Assembly Substrates

Silicon wafers with an 800 nm thermally grown SiO_2_ layer served as the base substrate. Interdigitated electrode arrays (width: 5 μm, gap: 1 μm) were fabricated using standard photolithography. Briefly, the wafers were spin-coated with a bilayer resist system comprising LOR 5B (lift-off layer) and S1813 (imaging layer), followed by soft baking at 180 °C (5 min) and 120 °C (2 min), respectively. UV lithography (SÜSS MA8, Garching, Germany) was used to pattern the electrodes, after which a Cr/Au (5 nm/40 nm) bilayer was deposited via electron-beam evaporation (EBE, AJA 2400-HY-UHV, Massachusetts, USA). Lift-off in dimethyl sulfoxide removed the excess metal and yielded well-defined metallic electrodes.

### 2.3. CNT Array Assembly Process

CNT assembly was performed on a probe station (FormFactor MPS 150, California, USA). A DC voltage was applied to the interdigital electrodes via probes using a precision source/measure unit (Keysight B2902A, California, USA). For each CNT array assembly experiment, 80 μL of CNT dispersion (optical absorbance spectra are shown in Figure S1) was dropped onto the substrate.

To investigate the effect of applied voltage on EDR-regulated assembly, DEP experiments were first conducted at DC biases of 3 V and 5 V using dispersion D1 (Figure S1), with the assembly time fixed at 150 s. Additional comparative experiments were then carried out under identical assembly time (150 s) to clarify the EDR-regulated mechanism based on a one-variable-at-a-time principle. Specifically, the effects of reducing the number of electrode pairs (from 50 to 5) and using a diluted CNT dispersion D2 (Figure S1) were examined separately. Based on these results, an optimized assembly was carried out using CNT dispersion D3 at a DC bias of 2.5 V using 100 electrode pairs for 150 s.

### 2.4. Characterization of CNT Arrays

The morphology of the CNT arrays was firstly characterized by scanning electron microscopy (SEM, Zeiss GeminiSEM 360, Oberkochen, Germany) to qualitatively/semi-quantitively evaluate their densities, alignment, and uniformity.

Cross-sectional transmission electron microscopy (TEM) was performed to confirm monolayer nanotube integrity. A 50 nm hafnium oxide (HfO_2_) capping layer was first deposited on the CNT arrays by atomic layer deposition (ALD, TEMAHf/H_2_O, 300 °C) to protect the fragile CNT structure during focused ion beam (FIB, Thermo Fisher Scientific Helios 5 CX, Massachusetts, USA) milling. Lamellae were then transferred to a spherical-aberration-corrected TEM (Thermo Fisher Spectra 300, Massachusetts, USA) for atomic-resolution imaging.

## 3. Definition of the EDR

To establish a framework for analyzing the evolution of CNT assembly during DEPs, we define the EDR as a kinetically determined spatial region near the electrodes where DEP-driven migration dominates over Brownian diffusion, enabling effective CNT capture. The formulation assumes a low-Reynolds-number, quasi-static system, neglecting convection and effects of the electric double layer, as well as considering only CNT migration prior to deposition.

Brownian motion describes the random motion of suspended particles arising from collisions with surrounding medium molecules [32], whose intensity is described by the translational diffusion coefficient, $D_{trans}$. For high-aspect-ratio CNTs, $D_{trans}$ can be expressed as

$$D_{trans}=\frac{k_{B}T}{6\pi \eta }\frac{2ln\left(l_{CNT}/d_{CNT}\right)-\gamma_{par}-\gamma_{per}}{l_{CNT}}$$

where η is the solution viscosity, $k_{B}$ is the Boltzmann constant, T is the ambient temperature, and $\gamma_{par}$ and $\gamma_{per}$ are hydrodynamic friction coefficients for motion parallel and perpendicular to the CNT long axis, respectively [33]. The friction coefficient γ is given by [34]

$$\gamma =\sum_{k}a_{k}\sigma^{-k}$$

$$\sigma =log(\frac{2l_{CNT}}{d_{CNT}})$$

In a non-uniform electric field, CNTs experience a DEP force arising from field-induced polarization. According to Pohl’s theory [35], the DEP force can be written as

$${\vec{F}}_{DEP}=\left(\vec{p}\cdot \nabla \right)\vec{E}$$

where $\vec{p}$ is the dipole moment induced by the local electric field $\vec{E}$. The dipole moment is given by

$$\vec{p}=v\tilde{\alpha }\vec{E}$$

where v is the CNT volume and $\tilde{\alpha }$ is the complex effective polarizability.

$$\tilde{\alpha }=\varepsilon_{m}{\tilde{f}}_{CM}$$

$${\tilde{f}}_{CM}=\frac{{\tilde{\varepsilon }}_{CNT}-{\tilde{\varepsilon }}_{m}}{\left({\tilde{\varepsilon }}_{m}+\left({\tilde{\varepsilon }}_{CNT}-{\tilde{\varepsilon }}_{m}\right)L_{1}\right)},\tilde{\varepsilon }=\varepsilon -i\frac{\sigma }{\omega }$$

$$L_{1}=d_{CNT}^{2}/l_{CNT}^{2}\left[\ln\left(\frac{2l_{CNT}}{d_{CNT}}\right)-1\right]$$

Here, ${\tilde{f}}_{CM}$ is the frequency-dependent Clausius–Mossotti factor, $L_{1}$ is the depolarization factor along the CNT long axis, and ${\tilde{\varepsilon }}_{CNT}$ and ${\tilde{\varepsilon }}_{m}$ are the complex permittivities of the CNT and medium, respectively. ε and σ denote the permittivity and conductivity, and ω is the angular frequency of the applied electric field [24,36,37]. The corresponding time-averaged DEP force associated with polarization along the CNT long axis is

$$\langle {\vec{F}}_{DEP}\rangle =\frac{\pi d_{CNT}^{2}l_{CNT}^{2}}{12}\varepsilon_{m}R\left[\frac{{\tilde{\varepsilon }}_{CNT}-{\tilde{\varepsilon }}_{m}}{\left({\tilde{\varepsilon }}_{m}+\left({\tilde{\varepsilon }}_{CNT}-{\tilde{\varepsilon }}_{m}\right)L_{1}\right)}\right]\nabla {\left|{\vec{E}}_{rms}\right|}^{2}$$

where ${\vec{E}}_{rms}$ is the root-mean-square electric field. Under viscous damping, the directed translational velocity of a CNT driven by the DEP force is

$${\vec{u}}_{DEP}=\frac{{\vec{F}}_{DEP}}{\gamma }$$

The characteristic time for Brownian diffusion over a length scale $L_{c}$ is

$$t_{Diff}∼\frac{L_{c}^{2}}{2D_{trans}}$$

The characteristic time required for a CNT to traverse the same distance under DEP-driven migration can be expressed as

$$t_{DEP}=\int_{s_{0}}^{s_{1}}\frac{d_{s}}{{\vec{u}}_{DEP}(s)}$$

where $s_{0}$ and $s_{1}$ denote the low-field and high-field positions along a migration path spanning the representative length $L_{c}$, and ${\vec{u}}_{DEP}(s)$ is the local DEP-driven velocity along this path. When the field variation over $L_{c}$ is limited, ${\vec{u}}_{DEP}$ can be approximated by a representative DEP-driven velocity, giving

$$t_{DEP}\approx \frac{L_{c}}{{\vec{u}}_{DEP}}$$

**Figure 1:**
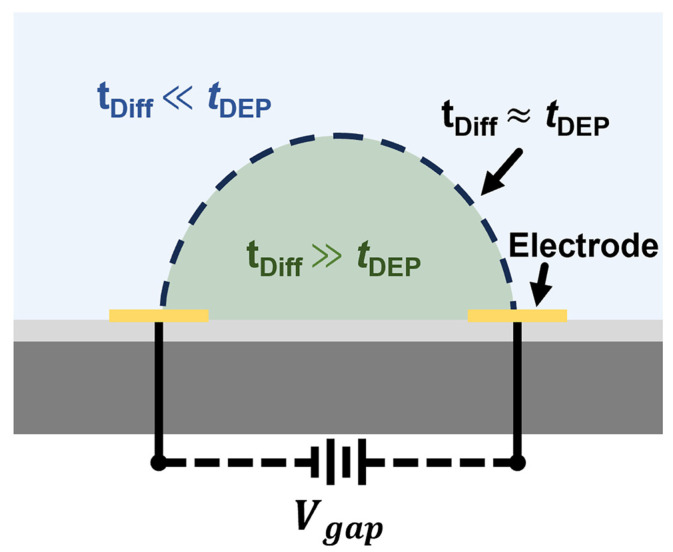
Schematic illustration of the EDR defined by the competition between DEP-driven transport and Brownian diffusion.

As illustrated in Figure 1, the EDR is defined as the spatial region where $t_{Diff}$ ≫ $t_{DEP}$, meaning DEP-driven deposition dominates over Brownian diffusion to sustain effective CNT capture. In contrast, when $t_{Diff}$ ≪ $t_{DEP}$, random diffusion dominates; therefore, CNTs cannot be captured or deposited effectively. The boundary region where $t_{Diff}$ ≈ $t_{DEP}$ indicates a kinetic transition zone between capture-dominated and diffusion-dominated CNT motion. In this sense, the EDR is used here as a kinetically defined process-state concept, whose evolution is inferred indirectly from electrical and modeling evidence rather than directly extracted as a geometric boundary from experiments.

## 4. DEP Condition–EDR–Array Framework for CNT Assembly

**Figure 2:**
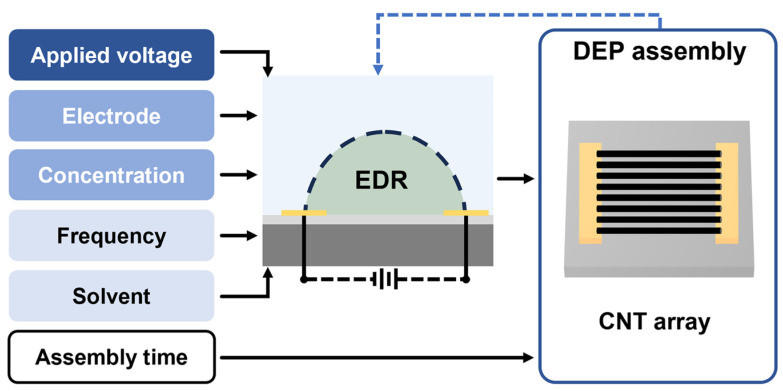
Schematic illustration of the DEP condition–EDR–array framework for CNT assembly.

Based on the definition of EDR, the CNT assembly process can be interpreted through a DEP condition–EDR–array framework, in which the EDR serves as an intermediate link between deposition conditions and the resulting array morphology (Figure 2). In contrast to prior self-limiting DEP descriptions that mainly focus on the eventual suppression of deposition [38], this framework uses the EDR to understand the dynamic evolution of effective CNT capture during assembly. Within this framework, DEP assembly can be understood as a dynamic process in which the EDR progressively evolves as CNT deposition continues. Under such assumptions, the major roles of the assembly conditions can be understood as follows. The applied voltage is fundamental for providing electric field within the EDR to deliver sufficient DEP driving force. Electrode geometry determines the EDR spatial distribution under the applied DEP voltage. CNT dispersion concentration, in turn, determines the number of nanotubes available for capture within the EDR. Notably, assembly time is not regarded as a deterministic parameter for CNT deposition in this framework, because the following analysis is based on quasi-static assembly conditions, under which the deposition state is determined primarily by the local EDR conditions at a given moment rather than by elapsed time itself. Therefore, the EDR provides a useful linkage for bridging both applied DEP conditions and assembly outcomes, providing a regulation approach for mitigating bundling during the densification of horizontal CNT arrays.

## 5. Result and Discussion

**Figure 3:**
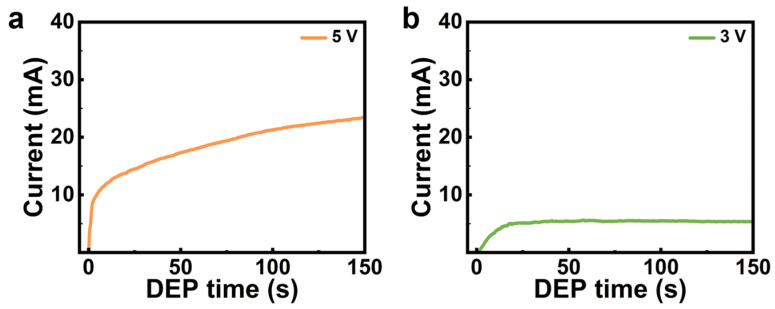
Typical circuit current monitoring during DEP assembly at DC biases of (**a**) 5 V and (**b**) 3 V using 50 pairs of parallel interdigitated electrodes.

To evaluate the effects of applied voltage on DEP assembly, experiments were performed at DC biases of 5 V and 3 V under otherwise identical assembly conditions. The real-time circuit current, which qualitatively indicates the deposition rate and bridging counts between electrodes, were employed to monitor the DEP process. At 5 V, the current increased immediately after the CNT dispersion was dropped onto the electrode region, and it continued rising throughout the 150 s assembly period, albeit with a gradually decreasing rate (Figure 3a). In contrast, at 3 V, the current increased at a rather lower rate compared to the 5 V case, and approached a stable plateau after ~20 s and remained constant thereafter (Figure 3b). These distinct temporal profiles indicate markedly different evolution of nanotube assembly under such two voltage conditions.

**Figure 4:**
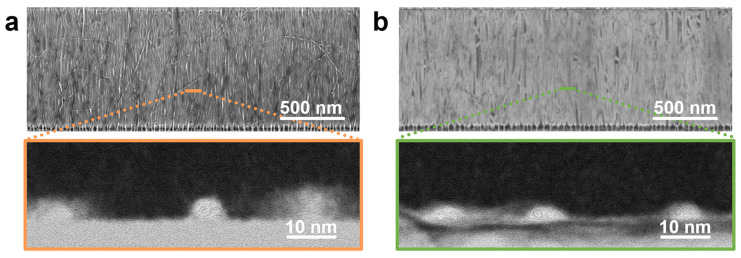
Morphological characterizations of CNT arrays assembled via DEPs with (**a**) 5 V DC and (**b**) 3 V DC for 150 s by SEM. Insets show the corresponding cross-sectional TEM images.

The SEM image in Figure 4a shows continuous CNT coverage with pronounced local bundling via DEP assembly at 5 V DC, which is further confirmed by the corresponding cross-sectional TEM. In comparison, the arrays assembled via 3 V DEP also exhibit continuous nanotube coverage but with substantially reduced bundling (Figure 4b). Cross-sectional TEM in Figure 4b further shows that the bundles formed at 3 V are smaller than those observed at 5 V. These results suggest that reducing the applied voltage changes the deposition behavior that suppresses excessive accumulation of deposited CNTs, thereby mitigating severe bundling.

**Figure 5:**
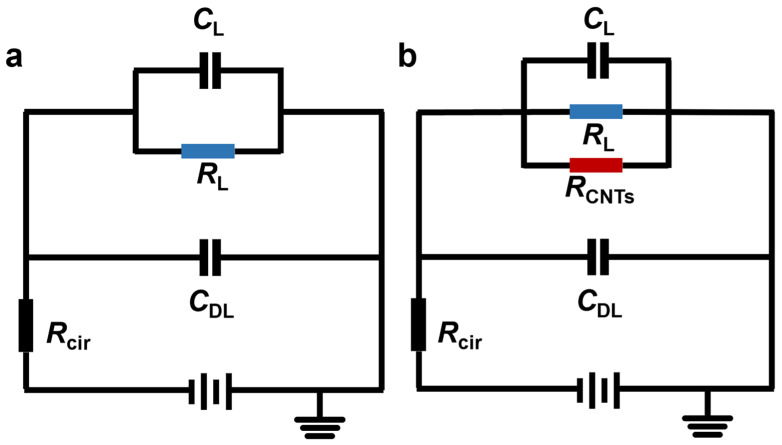
Equivalent circuit of DEPs (**a**) without CNT deposition, and (**b**) after CNT bridging across the interdigitated electrode pairs.

To interpret the above voltage-dependent assembly behaviors, the perspective of EDR evolution was employed based on the equivalent-circuit analysis of DEPs. Before CNT bridging occurs, the impedance across the electrode gap is dominated by the liquid medium. Owing to the low conductivity of xylene and its limited ion concentration, the electrode–liquid interface can be approximated as a capacitive branch in parallel with a liquid path [39,40]. Under a fixed applied voltage, the voltage drop across the gap is therefore determined mainly by the resistance of the liquid path together with the external circuit resistance (Figure 5a). As CNT bridges form, additional conductive pathways are introduced in parallel with the original liquid path (Figure 5b). These induced conductive components decrease the effective resistance of the gap, thus altering the effective DEP voltage partition within the equivalent circuit. As a result, the voltage across the electrode gap decreases progressively as the number of CNT bridges increases (see Supplementary Information Section S2 for details).

**Figure 6:**
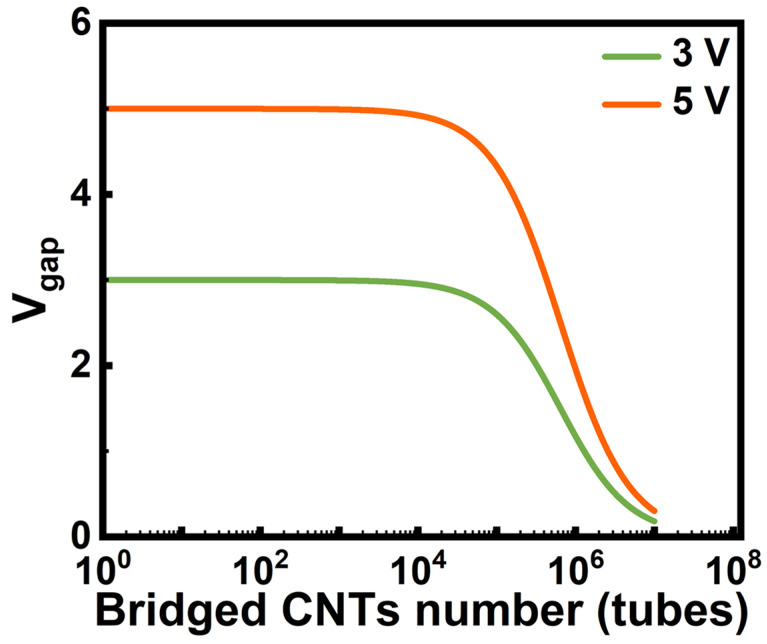
Model-predicted dependence of voltage $V_{gap}^{\prime }$ on the CNT-bridging number in DEPs with DC biases of 3 and 5 V, based on the equivalent circuit described in Supplementary Information Section S2.

Based on the equivalent-circuit analysis described above, Figure 6 shows the model-predicted dependence of the gap voltage, $V_{gap}^{\prime }$, denoting the voltage distributed only across the electrode gap, on the number of bridging CNTs (Supplementary Information S2). When there is only a limited number of CNT bridges, the induced conduction remains too weak to substantially alter the voltage partition within the DEP circuit, and $V_{gap}^{\prime }$ therefore decreases marginally. However, once a sufficient number of conductive pathways has formed, the induced conduction becomes significant, resulting in a rapid decay of $V_{gap}^{\prime }$. Since the local non-uniform DEP field is directly linked to $V_{gap}^{\prime }$, a reduction in $V_{gap}^{\prime }$ is expected to contract the EDR, thereby weakening the effective DEP-capture capability. Consequently, the circuit current approaches a plateau, indicating that further CNT bridging is suppressed. In this sense, EDR contraction provides a pathway that can reflect the subsequent evolution of CNT assembly based on circuit current monitoring.

Notably, the model predicts a lower $V_{gap}^{\prime }$ at 3 V than at 5 V under an identical number of bridging CNTs (Figure 6). This result indicates that the lower-voltage DEP condition exhibits more pronounced EDR contraction as conductive pathways accumulate, thereby leading to a capture-limited assembly. Such behavior is consistent with the observed current saturation at 3 V DEP, as shown in Figure 3b. In contrast, the slow decay of $V_{gap}^{\prime }$ for 5 V DEP allows for continuous CNT deposition, which increases the likelihood of local bundling.

**Figure 7:**
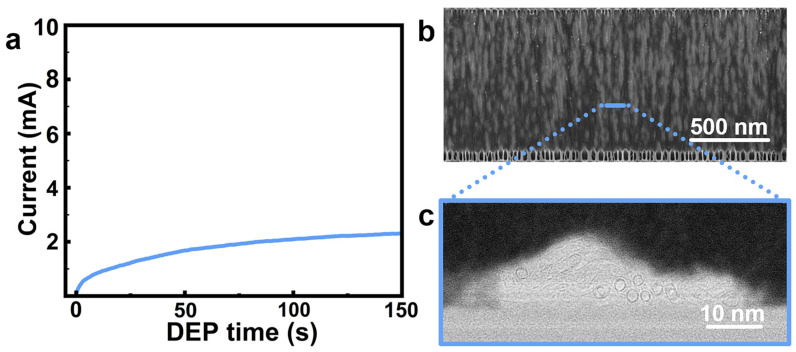
Effects of the number of parallel electrode pairs on the EDR-regulated DEP. (**a**) Circuit current monitoring during DEP assembly at a DC bias of 3 V using 5 parallel electrode pairs. (**b**) Typical SEM image of the assembled CNT arrays and (**c**) corresponding cross-sectional TEM image.

To further investigate the influences of DEP conditions on the EDR-regulated assembly, the number of electrode pairs and the concentration of CNT in dispersion are considered. When the number of parallel electrode pairs was reduced from 50 to 5 under a DC bias of 3 V, the circuit current increased rapidly at the initial stage but failed to reach a discernible plateau within the 150 s duration (Figure 7a). This sustained current increase illustrates continued accumulation of conductive pathways throughout the whole assembly period, suggesting that the deposition process remains unterminated under this condition because the contraction of the EDR is delayed. The SEM image in Figure 7b showed a pronounced CNT accumulation in the array, and the cross-sectional TEM (Figure 7c) characterization further revealed severe nanotube bundling. This can be attributed to the formation of fewer conductive CNT bridges under the 5-pair electrode configuration than the 50-pair base case within the same duration (Figure S2). The former provides a smaller cumulative conduction contribution and therefore slows the decay of $V_{gap}^{\prime }$, indicating a slower contraction of the EDR, which allows for sustained CNT capture and continuous deposition. These results verify that a sufficient number of electrode pairs is crucial for rapid EDR contraction to mitigate morphological deterioration of CNT arrays during the assembly densification.

**Figure 8:**
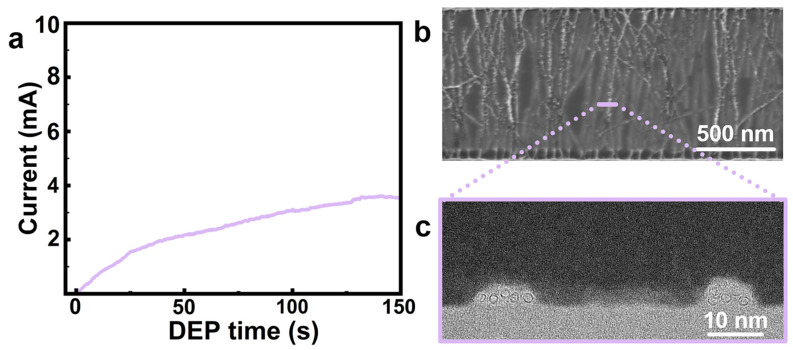
Effects of CNT dispersion on the EDR-regulated DEP assembly. (**a**) Circuit current monitoring during DEP assembly using diluted CNT dispersion D2 (Figure S2) with 50 electrode pairs at a DC bias of 3 V. (**b**) Typical SEM image of the assembled CNT arrays and (**c**) corresponding cross-sectional TEM image of CNT arrays.

The role of CNT dispersion concentration in EDR-regulated DEP assembly was further examined using a diluted dispersion D2 (absorption spectrum in Figure S1) with 50 pairs of electrodes under a DC bias of 3 V. As shown in Figure 8a, the current increased gradually without reaching an obvious plateau throughout the 150 s assembly period, indicating continued accumulation of conductive pathways under the diluted condition as a result of delayed EDR contraction. It is speculated that dilution in dispersion reduces the number of CNTs initially available within the EDR, thereby decreasing the bridging probability and slowing the EDR contraction (Figure S3). Furthermore, the smaller increase in current under the diluted condition illustrates that the total conduction contribution accumulated during such assembly was lower than that based on the undiluted condition. This interpretation is consistent with the reduced CNT coverage observed in the corresponding SEM image, as shown in Figure 8b. The cross-sectional TEM image (Figure 8c) further reveals more pronounced local bundling in the diluted case than in the undiluted case (Figure 4b).

**Figure 9:**
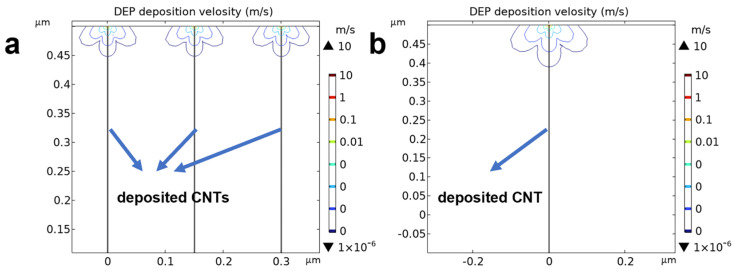
Simulated local deposition-velocity distribution of CNTs near pre-deposited nanotubes assembled via DEPs based on (**a**) undiluted dispersion and (**b**) diluted dispersion.

Finite-element simulations of the local deposition-velocity distribution of CNTs were employed to rationalize the array morphological differences between arrays assembled from diluted and undiluted dispersions under DEPs. Compared to the undiluted case (Figure 9a), DEP assembly with the diluted dispersion is expected to retain a higher $V_{gap}^{\prime }$ (Figure 9b), which maintains a broader and spatially heterogeneous DEP-driving field near the electrodes and deposited CNTs. Under such conditions, subsequent arriving CNTs are more likely to move toward localized high-field-gradient regions where pre-deposited nanotubes exist, thereby favoring bundle formation. Consequently, the arrays are globally sparse but exhibit pronounced bundling. These results support the view that a higher CNT dispersion concentration facilitates faster EDR contraction and thereby helps mitigate nanotube bundling.

**Figure 10:**
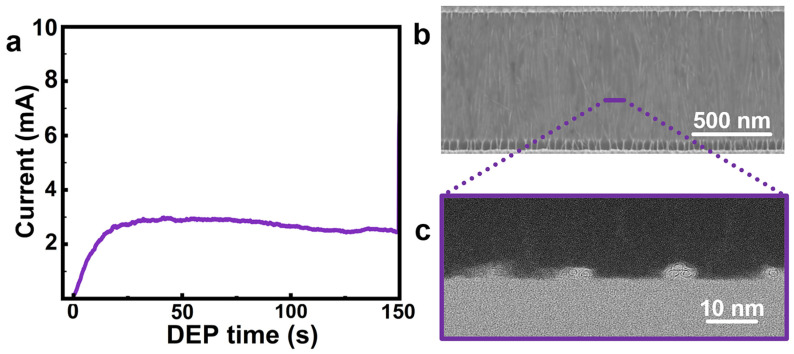
DEP assembly under the optimized EDR-regulated condition. (**a**) Circuit current monitoring during DEP assembly using CNT dispersion D3 (Figure S2) with 100 pairs of parallel interdigitated electrodes at a DC bias of 2.5 V. (**b**) Typical SEM image of the assembled CNT arrays and (**c**) corresponding cross-sectional TEM image of CNT arrays.

Guided by the EDR framework, a synergistically optimized DEP condition was designed and investigated using a DC voltage of 2.5 V, high-concentration CNT dispersion D3 (Figure S1), and 100 pairs of interdigitated electrodes. As shown in Figure 10a, the circuit current rose sharply at the initial stage and reached a plateau within approximately 15 s, indicating a rather quick and large conductive bridging. Notably, although a high-CNT concentration dispersion (D3) was used, the SEM image (Figure 10b) showed dense, continuous channel coverage without any obvious large bundles [28]. Cross-sectional TEM images (Figure 10c) revealed that the number of CNTs per unit width in the array was approximately 140 tubes μm^−1^. These results imply that EDR-regulated assembly is effective for guiding the design of DEP conditions to mitigate severe bundle formation during nanotube array densification.

Notably, the obtained arrays remain composed of small bundles (Figure 10c). One plausible explanation is that the present EDR framework does not explicitly account for interactions between deposited and incoming CNTs, which may alter local capture conditions and promote local bundling rather than ideal monolayer assembly. Future work shall therefore focus on such a combination of EDR with a self-limiting assembly strategy that suppress inter-tube attraction to achieve dense and horizontal monolayer array assembly [41,42].

## 6. Conclusions

In summary, we have developed an EDR-regulated DEP strategy for effectively suppress bundling during CNT array densification. By introducing the EDR concept, we reformulated the assembly process into a coherent and interpretable framework that links DEP conditions to the evolving effective CNT-capture capability and, consequently, to the final array morphology. Both the experimental observations and modeling results illustrate that conductive CNT bridging reduces the DEP gap voltage, contracts the EDR, and thereby limits CNT capture for continuous deposition. Through the synergistic optimization of applied voltage, electrode configuration, and CNT concentration, we obtained nanotube arrays in which the number of CNTs per unit width was approximately 140 tubes μm^−1^ and bundling was substantially mitigated.

It should be noted that the EDR is inferred indirectly rather than directly measured as an experimental boundary. In addition, although EDR regulation suppresses severe bundling, residual small bundles suggest that it alone is unlikely to obtain monolayer arrays, partly because the present framework does not explicitly account for inter-tube interactions after deposition. Future work focusing on a combination of EDR with a strategy that can suppress tube–tube attraction may enable the high-density monolayer assembly. Overall, this study illustrates that the EDR concept can provide an effective interpretation for understanding DEP deposition dynamics of CNT assembly and may pave a practical path for further pursuing dense, monolayer CNT array assembly.

## Data Availability

The original contributions presented in this study are included in the article/Supplementary Material. Further inquiries can be directed to the corresponding author.

## Supplementary Materials

The following supporting information can be downloaded at: https://www.mdpi.com/article/10.3390/nano16090512/s1, Figure S1. Typical optical absorbance spectra of the CNT dispersions used in this work. Figure S2. Schematic illustration of CNT-array assembly via DEPs with a reduced pair number of parallel interdigitated electrodes. Figure S3. Schematic illustration of EDR-regulated DEP assembly with undiluted and diluted CNT dispersions. Figure S4. Finite-element models for evaluating the concentration-dependent CNTs assembly behavior in DEPs using (a) low-concentration dispersion and (b) high-concentration one. Table S1. Parameters used in the equivalent circuit model. Table S2. Parameters and values used in the finite-element simulations. References [43,44,45,46] are cited in the supplementary materials.
