# Supplementary material for: A Strategy for Suppressing Bundling in Dielectrophoretically Assembled Carbon Nanotube Arrays
Source: Nanomaterials (Basel). 2026 Apr 24;16(9):512. doi: 10.3390/nano16090512 (PMC13164633; doi:10.3390/nano16090512)
Supplement: Supplementary file 1 [file nanomaterials-16-00512-s001.zip › nanomaterials-4279257-supplementary.pdf]

Supplementary Materials for

A Strategy for Suppressing Bundling in Dielectrophoretically Assembled Carbon Nanotube Arrays

Kai Wang[1,2], Rongbin Xie[1,2], Jianze Xiao[1,2], Yingnan Yang[1,2], Chaoqun Li[1,2], Zhengming Hao[1,2], Xiao Lei[1,2], Wenshan Li[1,2]*

[1] State Key Laboratory of Micro-Nano Engineering Science, School of Mechanical Engineering, Shanghai Jiao Tong University, Shanghai, 200240, China

[2] Micro-nano Engineering Sciences Research Center, School of Mechanical Engineering, Shanghai Jiao Tong University, Shanghai 200240, China

* Corresponding author: E-mail: wenshan.li@sjtu.edu.cn (*Wenshan Li*)

S1. Optical absorbance and nanotube concentration of CNT dispersions.

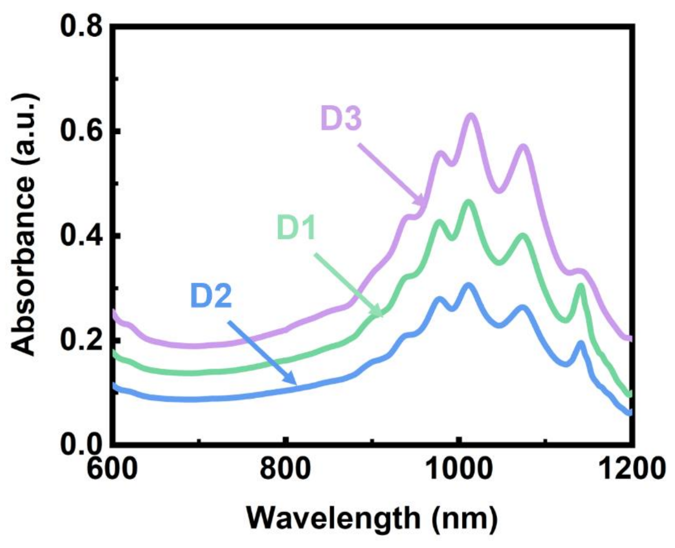

Figure S1. Typical optical absorbance spectra of the CNT dispersions used in this work.

S2. Equivalent circuit models.

To quantitatively analyze the evolution trend of the electric field during DEP assembly, we developed a simplified equivalent-circuit model to describe the expected trend of bridge-induced voltage redistribution during deposition. Therefore, the emphasis of the present analysis is placed on the decreasing trend of the effective gap voltage with increasing bridge number, rather than on the exact calculated values at any specific bridge count.

At the initial stage of DEP assembly, before any conductive CNT bridges are formed, the impedance between adjacent interdigitated electrodes is dominated by the liquid dispersion medium. The voltage across the electrode gap can be described by the corresponding V_{gap}:

$$V_{gap} = V_{app}\frac{R_L}{R_{cir}+R_L}$$

Where V_{app} is the applied voltage, R_L is the equivalent resistance of the liquid-pathway, and R_{cir} is the total resistance of the external circuit, including source and lead resistances.

As CNTs deposit and form conductive bridges, a parallel conductive branch associated with CNT bridging is introduced. The equivalent gap impedance R_{gap}, now comprising the liquid resistance R_L in parallel with the combined resistance of N bridging pathways, can be written as:

$$R_{gap} = \left(\frac{1}{R_L} + \frac{N}{R_{CNT}^{eq}}\right)^{-1}$$

$$R_{CNT}^{eq} = 2R_{con} + R_{CNT}$$

where R_{con} is the contact resistance between CNTs and the metal electrodes, R_{CNT} is the intrinsic resistance of an individual CNT, and N is the number of established bridging CNT pathways.

Consequently, the effective voltage across the electrode gap after bridge formation, V'_{gap}, becomes:

$$V'_{gap} = V_{app}\frac{R_{gap}}{R_{cir}+R_{gap}} = V_{app}\frac{R_L(R_{con}+R_{CNT})}{(R_{con}+R_{CNT})(R_{cir}+R_L)+NR_{cir}R_L}$$

This model reveals that CNT bridging decreases the effective impedance of the electrode gap, causing the actual voltage sustained across the gap to decay as the number of bridged CNTs increases. As a result, the local electric-field intensity and its gradient are progressively weakened during deposition rather than remaining constant throughout the assembly process. All key corresponding parameters are listed in the Table S1, which are representative values used in the simplified equivalent-circuit analysis and are not intended to represent the full experimental distribution of bridge resistances.

S3. Effects of electrode-pair number on EDR-regulated DEP assembly.

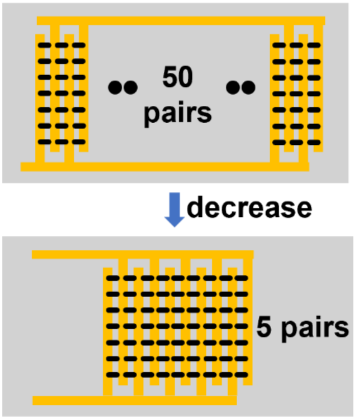

Figure S2. Schematic illustration of CNT-array assembly via DEPs with a reduced pair number of parallel interdigitated electrodes.

S4. Effects of CNT dispersion concentration on EDR-regulated DEP assembly.

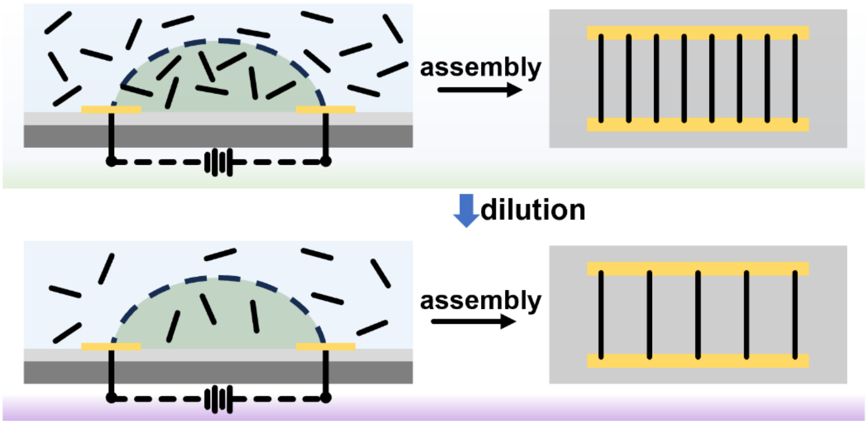

Figure S3. Schematic illustration of EDR-regulated DEP assembly with undiluted and diluted CNT dispersions.

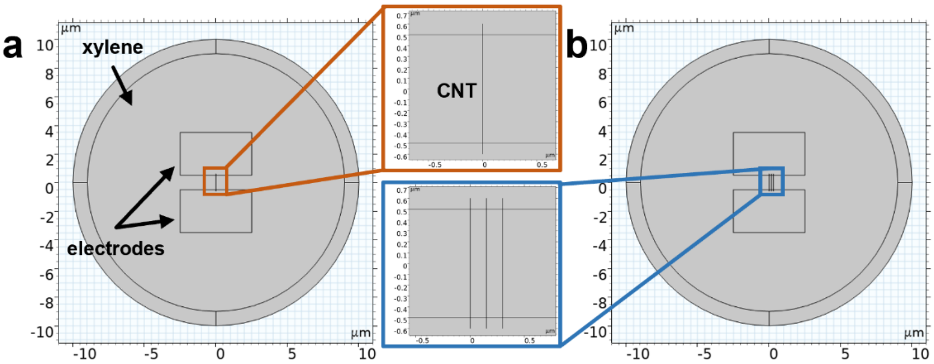

Figure S4. Finite-element models for evaluating the concentration-dependent CNTs assembly behavior in DEPs using (a) low-concentration dispersion and (b) high-concentration one.

Electric insulation boundary conditions were applied to the outer boundaries of the simulation domain. The two electrodes were set as the voltage terminal and ground, respectively, while electric-potential continuity was imposed across all internal interfaces. The parameters used in the simulations are summarized in Table S2.

Table S1 Parameters used in the equivalent circuit model.

Symbol	Physics	Value
V_{app}	Applied voltage (DC)	3 V/ 5 V
σ_L	Conductivity of Xylene	1.0×10^{-10} S/m
R_L	Liquid resistance	100 MΩ
R_{cir}	External circuit resistance [1]	82 mΩ
R_{CNT}	Intrinsic resistance of a CNT[2]	2.9 kΩ
R_{con}	Contact resistance between CNTs and electrodes [3]	50 kΩ

Table S2. Parameters and values used in the finite-element simulations.

Symbol	Physics	Value
L_{CNT}	CNT length	1.2 μm
D_{CNT}	CNT diameter	1nm
L_{ele}	Electrode length	5 μm
W_{ele}	Electrode width	3 μm
D_m	Xylene-covered region diameter	20 μm
V'_{gap}	voltage applied to electrode	3 V/1 V
T	temperature	300K
ε_0	Vacuum permittivity	8.854×10^{-12} F/m
ε_{CNT}	Relative permittivity of CNT[2]	$35\varepsilon_0$
ε_m	Relative permittivity of Xylene [4]	$2.27\varepsilon_0$
σ_{CNT}	Conductivity of CNT [2]	0.35 S/m
σ_m	Conductivity of Xylene [5]	1×10^{-12} S/m

Reference

[1] Li W, Hennrich F, Flavel B S, Dehm S, Kappes M and Krupke R 2021 Principles of carbon nanotube dielectrophoresis *Nano Res.* **14** 2188–206

[2] Li W, Hennrich F, Flavel B S, Kappes M M and Krupke R 2016 Chiral-index resolved length mapping of carbon nanotubes in solution using electric-field induced differential absorption spectroscopy *Nanotechnology* **27** 375706

[3] An L, Yang X and Chang C 2013 On contact resistance of carbon nanotubes *International Journal of Theoretical and Applied Nanotechnology* **1** 30–40

[4] Cannella W J 2007 Xylenes and ethylbenzene *Kirk-Othmer Encyclopedia of Chemical Technology* (John Wiley & Sons, Ltd)

[5] Mays H and Almgren M 1999 Temperature-dependent properties of water-in-oil microemulsions with amphiphilic triblock-copolymer. Part I: Dynamics, particle interactions, and network formation *J. Phys. Chem. B* **103** 9432–41
